# Bilateral Hydronephrosis From Retroperitoneal Fibrosis

**DOI:** 10.7759/cureus.12147

**Published:** 2020-12-18

**Authors:** Radhika Sheth, Devin Malik

**Affiliations:** 1 Internal Medicine, Henry Ford Health System, Jackson, USA; 2 Hematology/Oncology, Henry Ford Health System, Jackson, USA

**Keywords:** retroperitoneal fibrosis, chronic aortitis, steroids, obstructive uropathy

## Abstract

Retroperitoneal fibrosis (RPF) is a rare condition characterized by fibroinflammatory tissue infiltrating and compressing retroperitoneal structures. While mostly idiopathic (idiopathic retroperitoneal fibrosis or IRF), RPF is frequently associated with certain drugs, infections, and malignancies. It is thought to be immune-mediated because of response to steroids and RPF is commonly seen with other autoimmune diseases, especially IgG4-related disease (IgG4-RD). IRF is also a part of the chronic aortitis syndromes and the presence of aortic aneurysms is another characteristic of this disease. A 63-year old woman presented with left-sided flank pain. Computed tomography (CT) scan showed left hydronephrosis from compression of the ureter by a retroperitoneal mass. A thoracoabdominal aneurysm was also noted. A [^18^F]- fluorodeoxyglucose positron emission tomography (FDG-PET) scan showed hypermetabolism in the mass, with no abnormally increased activity noted elsewhere. Within four months, the mass enlarged to involve the right ureter as well, leading to right hydronephrosis. She required bilateral ureteral stents and aneurysm repair. Biopsy of the mass showed dense fibrosis with a mononuclear cell infiltrate. The histology of the aneurysm specimen showed chronic periaortic inflammation. Laboratory investigations were significant for elevated C-reactive protein (CRP) and erythrocyte sedimentation rate (ESR), with no evidence of monoclonal gammopathy. She was referred to the rheumatology clinic to receive steroid treatment for IRF. IRF commonly involves the ureters and is diagnosed on CT scans during a workup for obstructive uropathy. The treatment is high dose steroids, while in resistant cases, other immunosuppressants have been used. The presentation of a patient with IRF can commonly mimic that of urinary calculi and malignancy. While rare, IRF should not be forgotten when evaluating a patient for obstructive uropathy.

## Introduction

Retroperitoneal fibrosis is a rare disease characterized by chronic inflammation and fibrosis that infiltrates the retroperitoneal structures. It was first described by Albarran [1] in 1905 and then Ormond [2] in 1948, both describing ureteral obstruction from a retroperitoneal process. The incidence is estimated to be 1.3 per 100,000 persons, with a male: female predominance of 3.3: 1.0 [3]. It is most often idiopathic (in which case it is called idiopathic retroperitoneal fibrosis or IRF) but can also be seen in relation with certain drugs, malignancy, or infections. It is also sometimes seen in the presence of autoimmune conditions [4-6]. Recent literature [7,8] suggests that IRF is seen in the presence of aortic aneurysms as a part of the disease spectrum of chronic periaortitis (CP). The specific pathogenesis is poorly understood but it has been proposed that there is an exaggerated immune response triggered by atherosclerotic plaques in the aorta.

Because there is infiltration of the retroperitoneal organs, the clinical manifestations depend on the organ involved [9]. The diagnosis is made radiologically. However, as the appearance of IRF on imaging can mimic that of malignancy, a biopsy may be required to confirm the diagnosis. Most patients undergo a computed tomography (CT) scan as a part of the initial workup because their presenting symptoms are suspicious for obstructive uropathy. Magnetic resonance imaging (MRI) and [18F]-FDG PET scans are also done for further characterization of the mass. These scans frequently also reveal aortic aneurysms in these patients. The treatment is aimed at relieving the obstruction and slowing the progression of the fibrosis. Medical therapy with corticosteroids is considered first-line [10]. Other immunomodulators have been tried in patients who do not respond to steroids or relapse on steroids [11,12]. Here we describe a case that was initially diagnosed as malignancy and was later diagnosed correctly as retroperitoneal fibrosis on biopsy. The patient is currently undergoing treatment with high-dose steroids.

## Case presentation

A 63-year-old Caucasian female presented to the emergency department with worsening left-sided flank pain for five days. The pain wrapped around her left flank but did not radiate to the groin. She did not report symptoms of dysuria, urinary frequency, or hematuria. She did not report a fever, chills, or night sweats. Her past medical history includes only hypertension and depression. Her surgical history includes a spinal laminectomy and tubal ligation. She has never had urinary tract stones in the past.

On the initial examination, her vitals showed an oral temperature of 37.2 °C (98.9 °F), blood pressure of 117/72 mmHg, heart rate of 89 beats per minute, and a respiratory rate of 15 per minute. The cardiopulmonary exam was unremarkable. The abdominal exam was notable for some mild tenderness over the left upper and lower quadrants, without guarding or rigidity. Left-sided costovertebral angle tenderness was also noted. Initial laboratory tests showed a white blood cell count of 12 x 10^3^/ uL with neutrophilic predominance, hemoglobin 13.2 g/dL, platelet count of 283 x 10^3^/dL, serum creatinine 0.64 mg/dL, and glomerular filtration rate 96 ml/min/1.73 m^2^. The urinalysis only demonstrated a moderate amount of blood with 15 red blood cells per high power field. The liver function tests were within normal limits.

**Figure 1:**
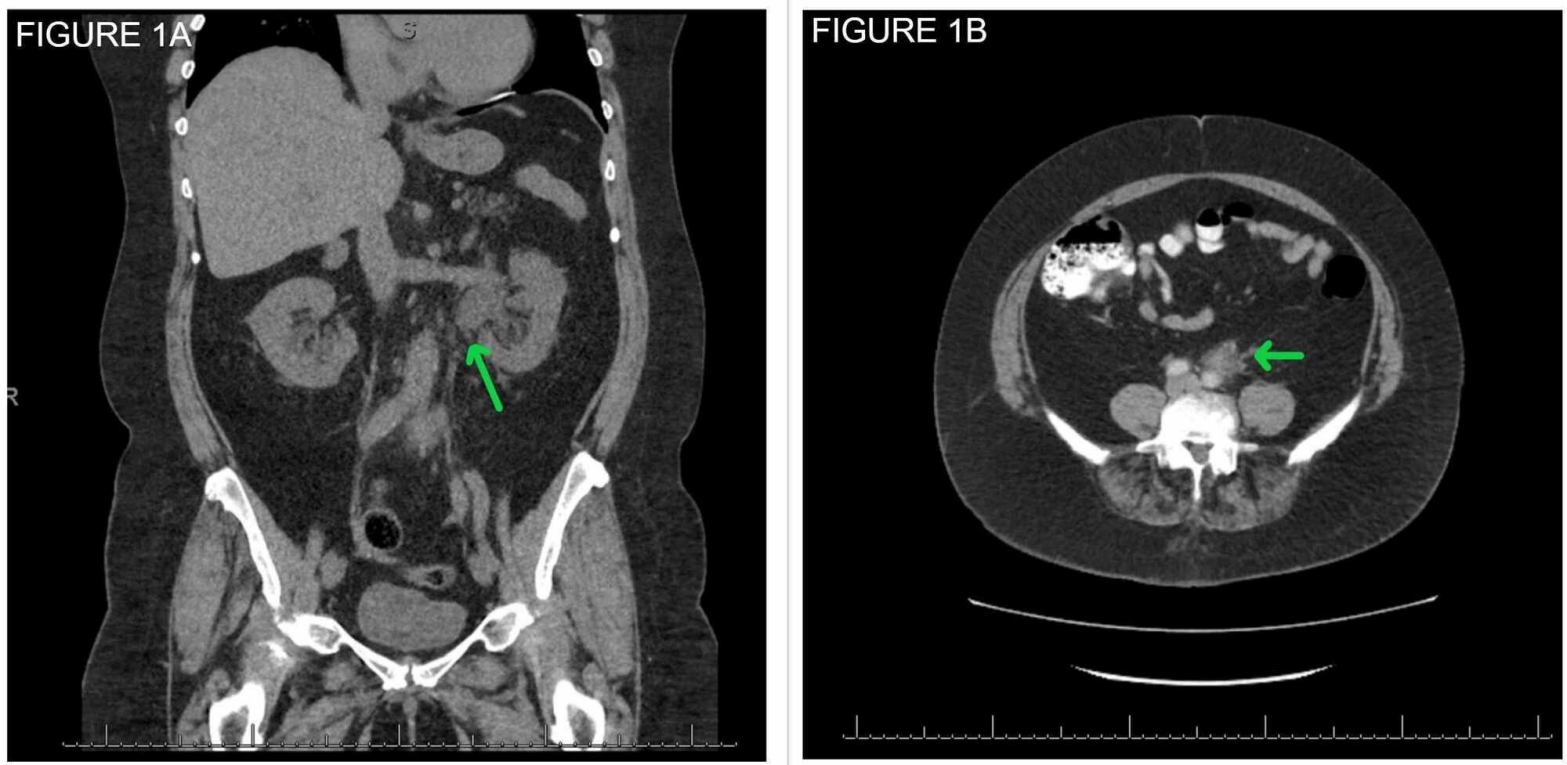
Initial CT scan of the abdomen and pelvis showing (A) Hydronephrosis on the left due to a retroperitoneal mass. Note the left-sided hydronephrosis and proximal hydroureter and (B) A 2.8 x 1.7 cm retroperitoneal soft tissue mass along the anterior margin of the aorta and abutting the mid-segment of the left ureter

**Figure 2:**
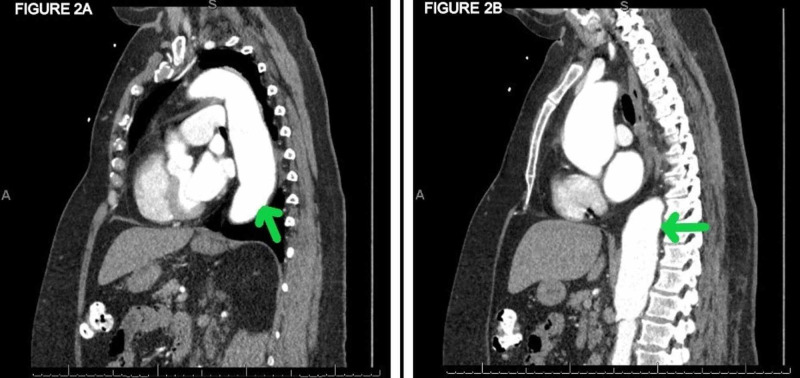
Initial CT abdomen demonstrating a (A) thoracic and (B) abdominal aortic aneurysm Note the fusiform aneurysm of the mid to distal descending thoracic aorta that measured up to 4.7 x 4.5 cm, and fusiform aneurysm of the suprarenal abdominal aorta measuring up to 4.2 x 4.1 cm.

A CT scan of the chest, abdomen, and pelvis showed mild left hydronephrosis and proximal hydroureter, but no calculus (Figure 1A). An ill-defined retroperitoneal soft tissue mass measuring 2.8 x 1.7 cm was seen along the anterior margin of the aorta and abutting the mid-segment of the left ureter (Figure 1B). She was also noted to have an ascending and descending thoracic aortic aneurysm (Figure 2A) and a proximal abdominal aortic aneurysm (Figure 2B). This was limited to the suprarenal abdominal aorta and the infrarenal part was normal in caliber.

 

**Figure 3:**
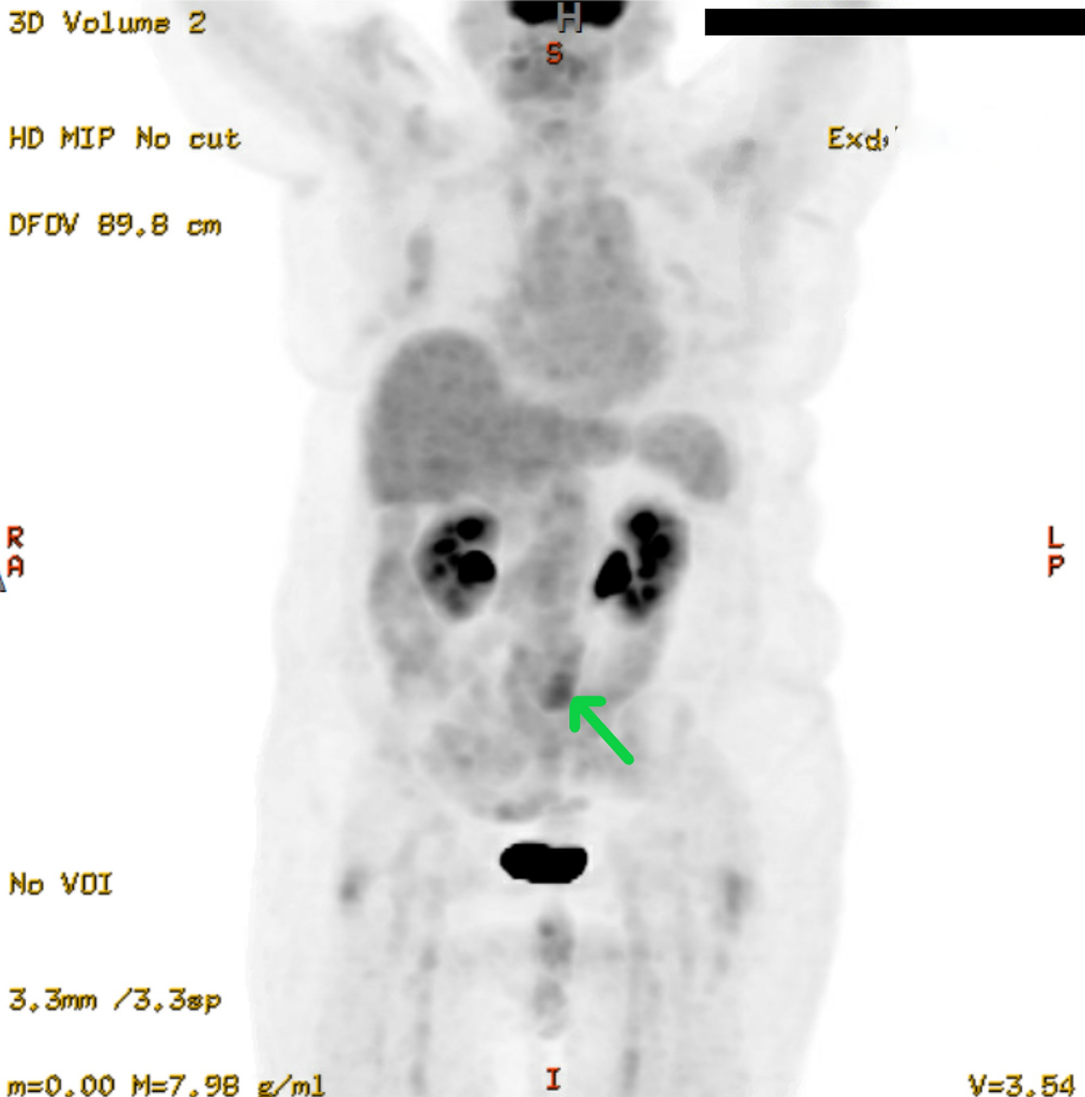
[18F]-FDG PET scan noting moderate hypermetabolism in the retroperitoneal mass

A ureteric stent was placed to relieve the obstruction after which there was a resolution of the hydronephrosis and hydroureter. She was discharged and later followed-up with the oncology clinic for evaluation of the mass. A positron-emission tomography (PET) scan was obtained that demonstrated a moderately hypermetabolic soft tissue mass encasing the distal left ureter (Figure 3). A surgical biopsy of the retroperitoneal mass was scheduled but was delayed due to the COVID-19 pandemic.

**Figure 4:**
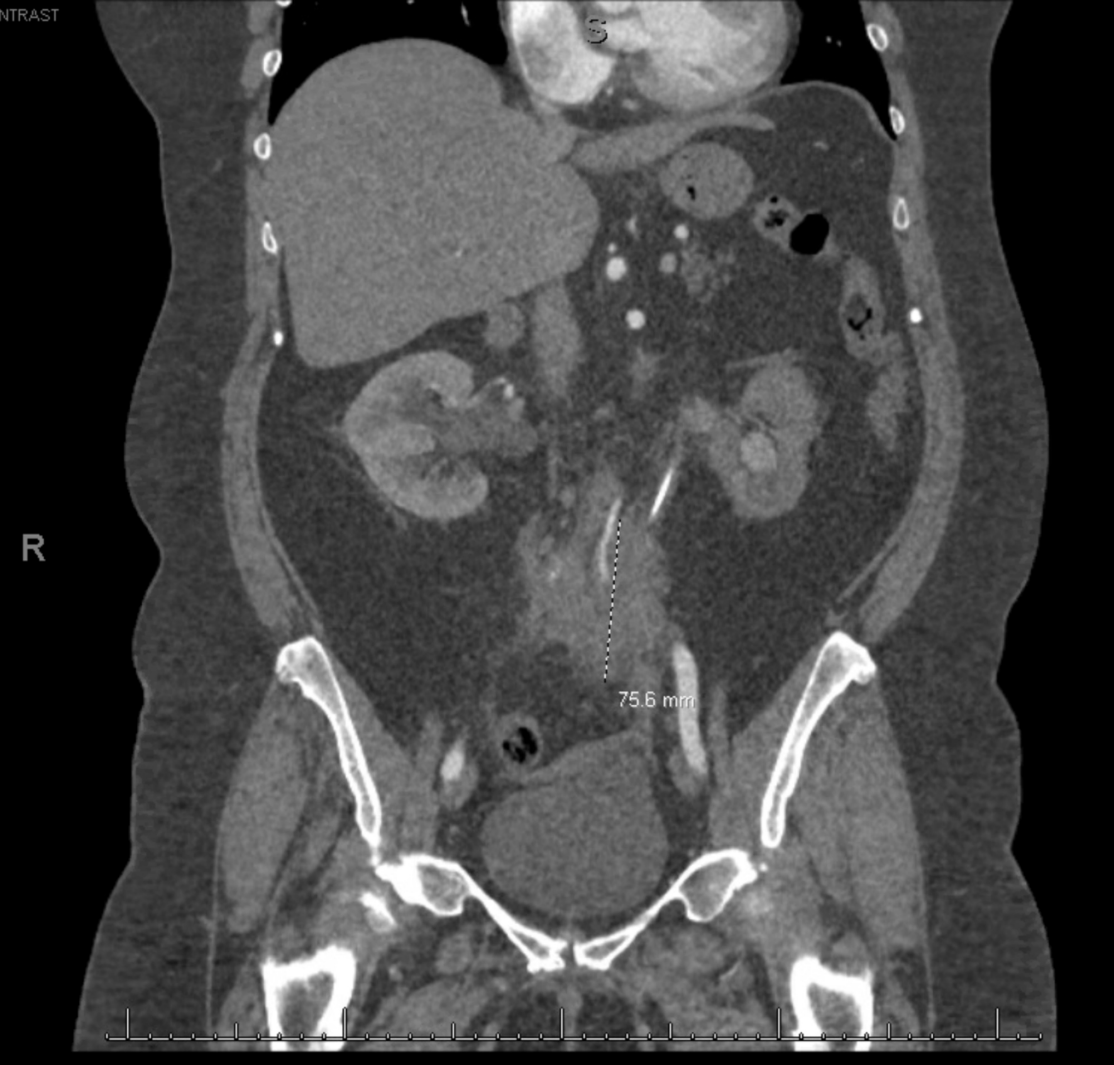
Subsequent CT scan, four months later, noting enlargement of the mass, now measuring 7.6 x 3.8 x 5.6 cm and encasing the right ureter causing mild right hydronephrosis.

**Figure 5:**
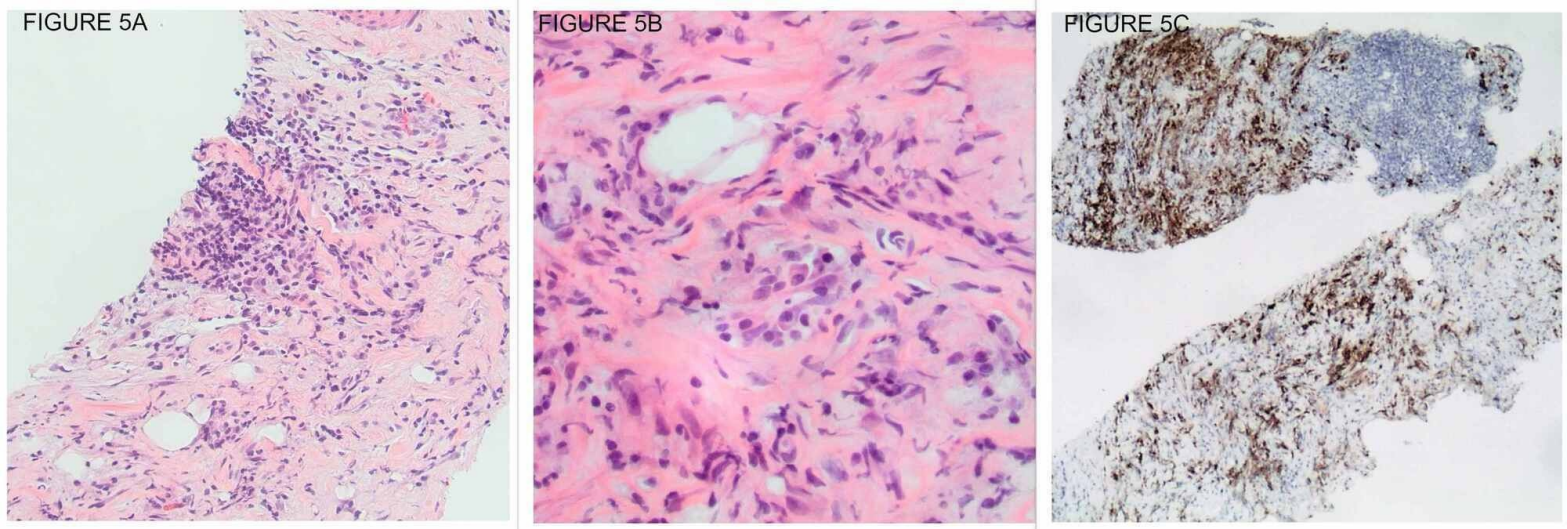
(A): Low-power view showing dense fibrosis with associated chronic inflammatory cells (H&E stain, 20x); (B): High-power view showing scattered plasma cells in a fibrotic background (H&E stain, 40x); (C): CD138 stain highlighting plasma cells (Immunoperoxidase, 40x magnification)

**Figure 6:**
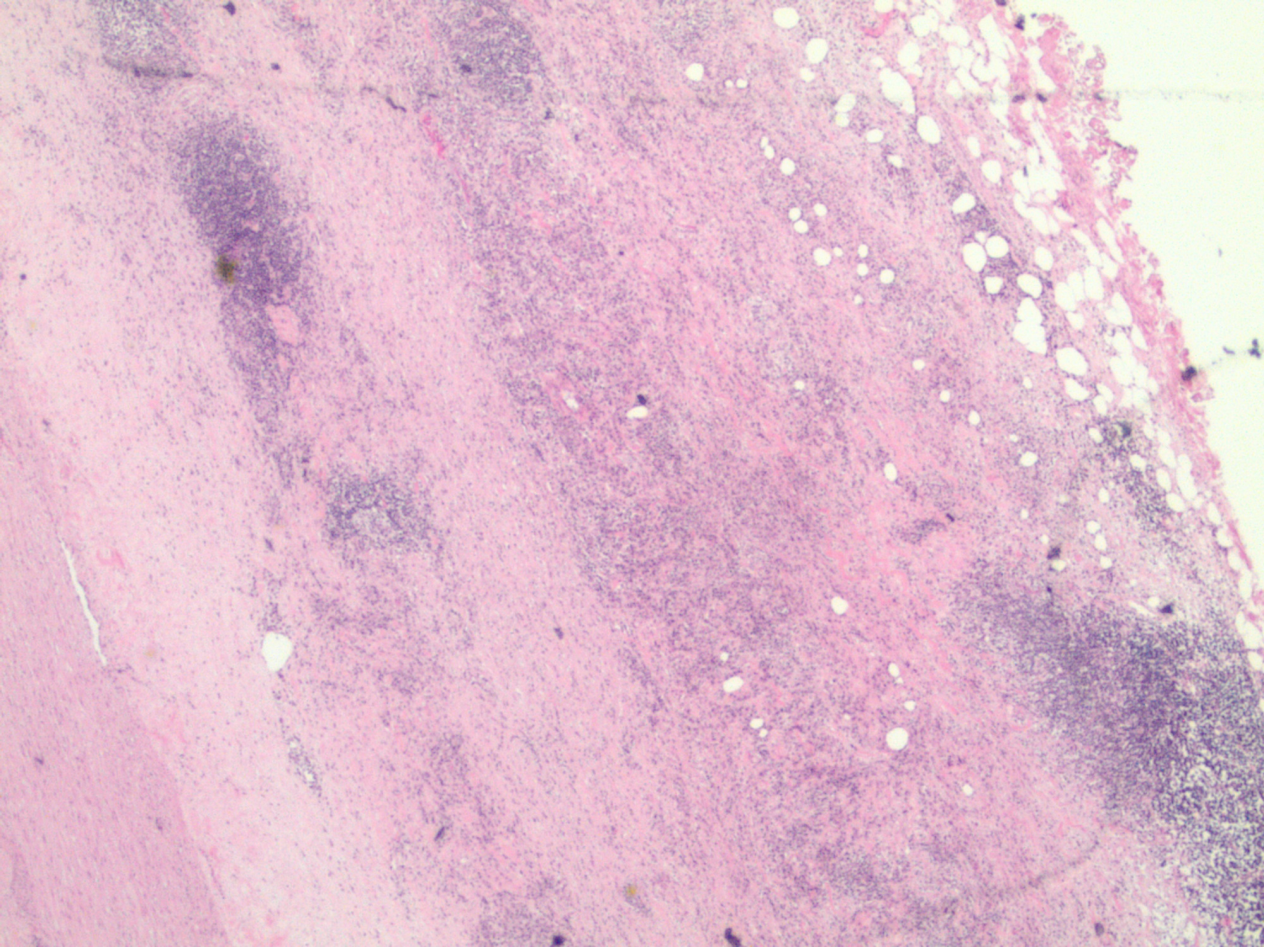
Low-power view of the aortic wall (tunica interna towards lower left corner) showing lymphoplasmacytic infiltrate in the tunica media and tunica adventitia, spilling into the periaortic soft tissue (H&E stain, 20x)

In the interim, within four months of the initial presentation, our patient developed new right-sided flank pain. A repeat CT scan demonstrated that the retroperitoneal mass had enlarged to 7.6 x 3.8 x 5.6 cm and was now encasing the right ureter causing mild right hydronephrosis (Figure 4). She had a right-sided ureteric stent placed this time to relieve the obstruction, and a CT- guided core biopsy was performed. The biopsy of the mass showed dense fibrosis with associated chronic inflammatory cells. CD3 stain highlighted scattered T-cell lymphocytes and a CD138 stain highlighted scattered plasma cells (Figure 5A-C). Staining was negative for CD30, Pax5, EMA, Alk-1, CD21, and CD23. FISH studies were negative for MDM2 amplification. She also underwent aneurysm repair and histology demonstrated periaortic chronic inflammation (Figure 6). The biopsy ruled out malignancy but with the presence of chronic inflammation on pathology, there was a suspicion for idiopathic retroperitoneal fibrosis. She was referred to the rheumatology clinic for further evaluation. 

 

Erythrocyte sedimentation rate (ESR) was 73 mm/hr and C-reactive protein (CRP) was 1.8 mg/dL. Antinuclear antibodies (ANA), anti-smooth muscle (anti-SM) antibodies, and anti-Ro/La antibodies were not detected. C3 and C4 complement levels were normal. Serum protein electrophoresis did not reveal any monoclonal gammopathy. Immunoglobulin G (IgG) subclass levels were also found to be within normal limits- IgG1: 510 mg/dL, IgG2: 363 mg/dL, IgG3: 87 mg/dL, and IgG4: 19 mg/dL. A diagnosis of idiopathic retroperitoneal fibrosis was made and she is currently undergoing treatment with high-dose steroids.

## Discussion

Retroperitoneal fibrosis is characterized by chronic inflammation and fibrosis infiltrating the retroperitoneal organs. It is most commonly idiopathic. However, certain drugs like ergotamine and etanercept, infections like tuberculosis, and malignancies like lymphomas, can also lead to retroperitoneal fibrosis [3,9,13] (Table 1). It can be present in conjunction with other autoimmune diseases like IgG4 related disease (IgG4-RD) [4]. Idiopathic retroperitoneal fibrosis (IRF) is now considered to be a part of the spectrum of chronic periaortitis (CP) which includes IRF, inflammatory abdominal aortic aneurysm (IAAA), and perianeurysmal retroperitoneal fibrosis (PRF) [7,8]. The latter two are histologically similar to IRF, but with the presence of aneurysms affecting the abdominal aorta, and sometimes even the thoracic aorta. Patients with IRF frequently have involvement of other organs [3,9] along with elevated acute phase reactants, suggestive of a systemic inflammatory process [14,15].

**Table 1:** Causes of Secondary Retroperitoneal FIbrosis

REPORTED SECONDARY CAUSES OF RETROPERITONEAL FIBROSIS	
Malignancy	Hodgkin’s and non-Hodgkin’s lymphoma, sarcoma, carcinoid tumors can cause secondary RPF. Histiocytic disorders like Erdheim-Chester can present with fibrosis around the kidney as well as RPF (“hairy kidney” on imaging. A biopsy is recommended in these cases for confirmation.
Asbestos and tobacco smoking	Studies have shown that exposure to asbestos and cigarette smoke are risk factors for RPF.
Medications	Ergot-derived medications like ergotamine and dopamine agonists like methysergide are believed to cause RPF due to increased serotonin levels. Etanercept, infliximab, and beta blockers have also been reported to be associated with RPF.
Infections	Tuberculosis can trigger fibrosis and other infections like actinomycosis and histiocytosis can present with RPF. Some experts recommend a biopsy in these cases.
Radiation	Radiation as part of treatment for certain cancers can also cause RPF.

The specific pathogenesis is poorly understood. However, it has been proposed that there is an exaggerated immune response triggered by atherosclerotic plaques in the aorta. There is a proliferation of CD4+ cells in the aortic wall and retroperitoneum. These cells secrete interleukin-6 (IL-6) which causes B cells and fibroblast proliferation. The infiltrating B cells can expand to IgG4- producing plasma cells as seen in IRF in IgG4-RD. Exposure to asbestos and cigarette smoke are major risk factors [9]. There is also an association with HLA-DRB1*03 [5], which is linked to other autoimmune conditions like Hashimoto’s thyroiditis and type 1 diabetes mellitus [6].

While the clinical manifestations can vary, the most common presenting symptoms are unilateral or bilateral flank pain resembling ureteric colic and abdominal pain. This is because the fibrosis can encompass the abdominal aorta and can contiguously involve the ureters as well, causing ureteric obstruction and renal insufficiency. Patients may also present with pain radiating to the groin, testicular pain, or varicocele. Some patients report constitutional symptoms of malaise, anorexia, weight loss, nausea, and fever. There may also be lower extremity edema due to venous compression and claudication symptoms due to arterial compression. There can be involvement of extra retroperitoneal organs like the pancreas, salivary glands, and orbits, and this is frequently seen in patients with IgG4-RD [3,9].

Laboratory investigations may show acute kidney injury depending on the degree of ureteric obstruction. Urinalysis findings are usually benign. However, hematuria and proteinuria can be present. A majority of the patients have elevated inflammatory markers, specifically the erythrocyte sedimentation rate (ESR) and C-reactive protein (CRP) [14,15]. Titres for antinuclear antibodies (ANA) and anti-neutrophil cytoplasm antibodies (ANCA) may also be positive, especially in the presence of other autoimmune conditions [6]. Patients with IRF associated with IgG4-RD have an elevation of IgG4 subclass [4].

Given the presentation, patients with IRF frequently undergo an initial workup for obstructive uropathy, as seen in the case of our patient. They may initially have a renal ultrasound to rule out urinary tract obstruction. The ultrasound may visualize the fibrosis as an ill-defined hypoechoic mass compressing one or both ureters and causing hydronephrosis. A CT scan with contrast is the preferred imaging modality that shows soft tissue encasing the retroperitoneal structures, with or without localized lymphadenopathy. The CT scan may also show the presence of an aortic aneurysm. MRI and [18F]-FDG PET scans are also done sometimes, especially to rule out a malignant process. [18F]-FDG PET scans have emerged as a promising new imaging technique for IRF. On PET scans, the retroperitoneal tissue appears hypermetabolic. PET scans can also be used to look for extra-retroperitoneal involvement and to monitor response to treatment [16]. A study by Fernando et al. where 78 patients underwent a PET scan suggested that PET scans may reduce the need for invasive biopsies in IRF, and there may be a correlation of FDG avidity of the mass with responsiveness to treatment [17].

Malignancies like lymphoma, infections like tuberculosis, and infiltrative diseases like Erdheim-Chester disease should also be included in the differential. A diagnostic biopsy is recommended if there is suspicion of malignancy or infection. The histology in IRF shows fibrous tissue composed of type I collagen fibers arranged around the vessels, with a large number of myofibroblasts and chronic inflammatory infiltrate. This infiltrate consists of lymphocytes, macrophages, and plasma cells with conspicuously absent neutrophils. It is typically a nodular arrangement or aggregated perivascularly with a core of CD20+ B cells and a peripheral rim of CD4+ and CD8+ T cells. Plasma cells positive for IgG4 may also be observed and if the proportion of these cells is more than 40%, the disease is said to be IgG4- related [12]. The presence of granulomas may be seen in tuberculosis, and lipid-laden macrophages (“foamy histiocytes”) in Erdheim- Chester disease.

The treatment of IRF involves relieving the obstruction caused by the fibrosis and limiting further spread. As most patients present with urinary obstruction, urology or surgical services may need to be involved for placement of ureteral stents or nephrostomy tubes. Previously open or laparoscopic surgical interventions like ureterolysis were commonly performed, but more conservative interventions are now preferred. Rarely, if the obstruction is mild with no significant renal impairment, the patients can be monitored on medical therapy alone.

If an inciting factor like a drug or an infection is found, definitive treatment is aimed at the removal of the offending drug or treating the infection. While there is no standard treatment or guidelines, glucocorticoids are considered to be first-line therapy for idiopathic disease. High dose steroids (1- 1.5 mg/kg/day) for up to four weeks, tapered to a maintenance dose for six-nine months have been shown to achieve rapid resolution of symptoms and decrease in the size of the mass on imaging [10].

Failure to respond to steroids may require surgical intervention. It should also prompt a second look to confirm the diagnosis and rule out a malignancy that may have been missed initially. Immunosuppressants like methotrexate, mycophenolate, cyclosporine, cyclophosphamide, and rituximab- either alone or in combination with steroids- have also been used with variable success, specifically in steroid-resistant cases [11,12,18,19]. Given the expression of IL-6 inciting the fibroblastic response, IL-6 has emerged as a new target for therapy. Tocilizumab, an IL-6 inhibitor, has been used successfully in some refractory cases [20]. While monitoring with serial imaging and ESR and CRP is suggested, studies by Magrey et al. [14] and Pelman et al. [15] have shown that acute phase reactants are poor predictors of response to therapy. Depending on the presence of the aortic aneurysms, the patients need close monitoring of the size with ultrasound imaging and may require surgical repair if indicated. Once in remission, patients with IRF should be monitored closely for relapse. In a case series by van Bommel et al. where 24 patients with IRF treated with steroids were followed for 1 year, 22 patients reported resolution of symptoms. However, relapse rates were found to be as high as 72% [10]. Patients may also have multiple relapses and require steroid-sparing therapy.

In our patient’s case, initially, she had a workup for obstructive uropathy from kidney stones and the imaging showed a retroperitoneal mass compressing the ureters. The case was initially diagnosed as malignancy and the patient was referred to the oncology service. The COVID-19 pandemic delayed further investigations and the mass enlarged in size. When the biopsy was finally performed, it ruled out malignancy, and finally, the diagnosis of idiopathic retroperitoneal fibrosis was made.

## Conclusions

In summary, IRF can have protean manifestations and, while uncommon, it is prudent to consider it as a part of the differential when evaluating obstructive uropathy. The treatment guidelines have not been well established, but high-dose corticosteroids appear to be effective in most cases. In refractory cases, steroid-sparing drugs and other immunomodulators have shown some promise. Even though it follows a chronic relapsing course, patients with IRF generally have good outcomes.
